# Reconciling Homeostatic and Use-Dependent Plasticity in the Context of Somatosensory Deprivation

**DOI:** 10.1155/2015/290819

**Published:** 2015-03-18

**Authors:** John J. Orczyk, Preston E. Garraghty

**Affiliations:** ^1^Department of Psychological and Brain Sciences, Indiana University, Bloomington, IN 47405, USA; ^2^Program in Neuroscience, Indiana University, Bloomington, IN 47405, USA

## Abstract

The concept of homeostatic plasticity postulates that neurons maintain relatively stable rates of firing despite changing inputs. Homeostatic and use-dependent plasticity mechanisms operate concurrently, although they have different requirements for induction. Depriving central somatosensory neurons of their primary activating inputs reduces activity and results in compensatory changes that favor excitation. Both a reduction of GABAergic inhibition and increase in glutamatergic excitatory transmission are observed in input-deprived cortex. Topographic reorganization of the adult somatosensory cortex is likely driven by both homeostatic and use-dependent mechanisms. Plasticity is induced by changes in the strengths of synaptic inputs, as well as changes in temporal correlation of neuronal activity. However, there is less certainty regarding the *in vivo* contribution of homeostatic mechanisms as *in vitro* experiments rely on manipulations that create states that do not normally occur in the living nervous system. Homeostatic plasticity seems to occur, but more *in vivo* research is needed to determine mechanisms. *In vitro* research is also needed but should better conform to conditions that might occur naturally *in vivo*.

## 1. Introduction

The hypothesis of homoeostatic plasticity postulates that neurons defend a relatively constant rate of firing even as individual synaptic inputs undergo changes in their strength (for reviews of homeostatic plasticity see [1, 2]). Homeostatic plasticity predicts that prolonged excitation will promote inhibitory adaptations in neurons, while prolonged activity deprivation will promote adaptations that facilitate excitation. On the surface, these predictions conflict with well-established manifestations of use-dependent plasticity that demonstrate increases or decreases in synaptic strengths with either high or low frequency input activation. Unlike mechanisms associated with homeostatic plasticity, use-dependent plasticity drives changes in synaptic strength at specific synapses. Presumably, then, if these two phenomena are concurrently operating in the intact nervous system, neurons that undergo changes in the strengths of specific synapses via use-dependent plasticity can still maintain a global balance between excitation and inhibition only if the strengths of other synaptic connections are adjusted to compensate for the use-dependent changes. Here, we will attempt to reconcile homeostatic and use-dependent plasticity in the context of somatosensory cortical reorganization following the loss of primary input. Reorganization in deprived somatosensory cortex is likely driven by both homeostatic and use-dependent mechanisms. However, there is less certainty regarding the* in vivo* contribution of homeostatic mechanisms as* in vitro* experiments rely on manipulations that create states that do not normally occur in the living nervous system.

## 2. *In Vivo* Homeostatic Plasticity Experiments

Homeostatic plasticity has putatively been demonstrated* in vivo* in the mouse barrel cortex, visual cortex, and gerbil auditory cortex. In the adult mouse barrel cortex, chronic stimulation of a mystacial whisker follicle for 24 h results in the insertion of both excitatory glutamatergic and inhibitory GABAergic receptors on dendritic spines. While Hebbian theory predicts a use-dependent strengthening of stimulated synapses, if left unchecked, this positive feed-forward mechanism would drive neuronal networks past physiological limits of excitability. Thus, the insertion of inhibitory synapses acts as a means to maintain physiological limits of excitability and preserve neuronal homeostasis. In other words, the action of chronically stimulating neurons in the barrel cortex for 24 h led to the reaction of inserting more inhibitory GABAergic synapses in order to oppose chronic excitation and maintain relatively stable firing rates within the neural network. Four days after stimulation, however, the GABAergic synapses on dendritic spines remained while the density of excitatory synapses returned to prestimulation levels [3]. This finding would seem to run counter to predictions based solely on homeostatic mechanisms as one would expect that the activity levels of these neurons at this time would be lower due to the increased inhibitory input. Perhaps, if the investigators had waited longer for their final assay, they may have discovered that the GABAergic synapses had also been retracted. In any event, the research presented would appear to be consistent with the operation of homeostatic mechanisms after one day of chronic stimulation, but the findings at day four do not seem to be compatible with homeostatic theory in any straight-forward way. These authors also suggest that the latter findings are suggestive of a “trace” of the chronic stimulation (cf. [4, 5]). Such “traces” would also seem to pose a challenge for purely homeostatic mechanisms.

Visual deprivation (VD) elicits homeostatic plasticity in both mice visual cortex and barrel cortex. Following 7 days of VD through either dark exposure or binocular enucleation, mEPSCs from AMPARs were pharmacologically isolated in slices of visual and barrel cortex. VD increased AMPAR mEPSCs amplitudes in visual cortex while decreasing them in the barrel cortex. The decrease in the barrel cortex was not associated with changes in whisking behavior. Interestingly, VD through the use of eyelid sutures, which allows for the transmission of diffuse light to the retina, was insufficient to increase mEPSC amplitude in the visual cortex but did decrease the amplitude in the barrel cortex. The results demonstrate that VD results in both unimodal and cross-modal homeostatic plasticity in sensory systems. However, plasticity in each modality occurs independently of each other and relies on different sensory requirements [6].

Monocular deprivation (MD), accomplished through eyelid suturing, also leads to homeostatic changes in synaptic strength. In binocular cortex, neurons that receive inputs from both eyes strengthen open eye responses and weaken deprived eye responses over a period of 5 days of MD. This process may rely on either homeostatic or Hebbian forms of plasticity. Strengthening open eye responses allows neurons to maintain a constant rate of firing when deprived of some of their inputs. MD also disrupts the correlation between visual stimulation and binocular neuron firing for the deprived eye. In Monocular cortex, 5 days of MD lead to a strengthening of responses to the deprived eye when the sutures are removed. These neurons receive input from only the deprived eye and cannot adjust their available inputs to maintain constant firing rates. Instead, they scale up the strength of their connection to the deprived eye. Increased sensitivity to the deprived eye may help monocular neurons defend their activity levels [7]. One can note that other experiments have been reported that attempted to isolate the effects of visual deprivation* per se* from any contributions of binocular competitive interactions [8–11]. In none of these cases were deprived neurons protected from deleterious effects of deprivation. It should be further noted, however, that these latter studies were conducted in the developing visual system. Perhaps, homeostatic mechanisms had not matured completely and, thus, were not completely functional in the subjects employed in these experiments.

Amyloid-beta (A*β*) may play an important role in homeostatic plasticity in the visual cortex. A*β* is produced in an activity-dependent manner. Overproduction or exogenous application of A*β* can induce AMPA receptor endocytosis, weakening synaptic transmission. Mice lacking the major neuronal secretase BACE1 display higher basal rates of synaptic transmission in the visual cortex. These mice also fail to scale up strengthens of visual cortical synapses in response to VD. A*β* appears to play a role in homeostatic regulation of synaptic transmission [12].

Homeostatic plasticity has been demonstrated* in vivo* following cochlear ablation in postnatal gerbils, resulting in sensorineural hearing loss (SNHL). This procedure, which deprives the auditory cortex of its principle input, produces enhanced excitability in the auditory cortex. Thalamocortical brain slices were prepared and A1 neurons in the auditory cortex were stimulated by an electrode implanted in the thalamic ventral medial geniculate nucleus (MGv). The average resting membrane potential of A1 neurons from gerbils with SNHL was slightly depolarized in comparison to control A1 neurons. The firing patterns of A1 neurons from gerbils with SNHL were also altered with a 30% increase in sustained firing neurons, a 13% reduction of adapting neurons, and a complete absence of onset firing neurons in comparison to the firing patterns of neurons in control auditory cortices. Removing the principle driving input resulted in changes in neuronal firing patterns that favor sustained firing. Voltage clamped preparations revealed EPSCs of extended durations in A1 neurons from gerbils with SNHL. The use of the NMDAr2B subunit-specific receptor antagonist ifenprodil produced greater reductions in EPSP durations in A1 neurons from gerbils with SNHL, suggesting increases in NMDAr2B contributions to the EPSP. Immunostaining revealed increases in NMDAr2B expression in both pre- and postsynaptic locations. The NMDAr2B subunit enhances receptor sensitivity to glutamate and prolongs the opening time. Increases in excitatory transmission were accompanied by decreased inhibition. IPSCs, measured in the presence of AMPA and NMDA antagonists, were smaller in amplitude in SNHL A1 neurons. These adaptations serve to enhance excitability in input deprived auditory cortex [13]. Interestingly, an increase in the ratio of NMDAr2A to NMDAr2B units occurs developmentally at the end of the critical period in the visual system. This change can be delayed by sensory deprivation in the visual system, paralleling the increase in NMDAr2B seen in auditory cortex. Thus, one could argue that A1 neurons from gerbils with SNHL have reverted to an immature state in this particular regard in response to the sensory deprivation. We have argued previously that neurons in deprived regions of somatosensory cortex of adult monkeys pass through a phase that we characterized as developmental recapitulation [14, 15]. Perhaps, “turning the clock back” is an inherent neural response to input deprivation.

*In vivo*, it is often unfeasible to make clear distinctions between homeostatic and use-dependent plasticity, although both are experience-dependent. An example is plasticity resulting in the strengthening of intact inputs when others are eliminated. Neurons in the rat barrel cortex are arranged in cellular aggregates that respond to a principal whisker. However, inputs overlap such that neighboring whiskers can also activate barrels, although to a lesser extent than the principal whisker. When all but two neighboring whiskers on one side of the rat face are trimmed, responses from the neighboring trimmed whiskers are decreased. However, the response to stimulation of the intact whiskers, both the one neighboring whisker and the principal whisker, increases [16]. This example of plasticity resembles homeostatic plasticity because there is strengthening of inputs in response to activity deprivation. The strengths of available inputs are adjusted towards maintaining a consistent rate of firing for the barrel deprived of input from the majority of its neighboring whiskers. However, this example of plasticity also resembles use-dependent plasticity because it is driven by changes in the temporal pairing of inputs. When all but two neighboring whiskers are clipped, sensory input for the intact whiskers is frequent and occurs in synchrony while input from the clipped whiskers is infrequent and does not occur in synchrony with the intact whiskers. This example of plasticity may be considered a rudimentary example of cortical reorganization in the somatosensory cortex when sensory inputs are altered. As will become apparent, focusing on drawing clear distinctions between homeostatic and use-dependent plasticity obscures the actuality that these types of plasticity occur in concert* in vivo*.

## 3. Somatosensory Deprivation: Cortical Reorganization as Homeostatic Plasticity

Somatotopic reorganization in the primary sensory cortex following the loss of peripheral input through amputation or deafferentation has been observed in rodents (e.g., [17, 18]), nonhuman primates (e.g., [19–24]), and humans (e.g., [25, 26]). Processes underlying reorganization include the immediate unmasking of latent inputs as well as expansion of adjacent sensory inputs into the cortical area deprived of peripheral input. In humans, reorganization of the somatosensory cortex following limb amputation may contribute to the development of phantom pain in the amputated limb [25, 26]. It would appear that homeostatic mechanisms could play important roles in these manifestations of reorganizational plasticity. That is, as neurons lose their principle driving input due to the loss of sensory input, a homeostatic balance of excitability and inhibition could be maintained only if they find new inputs.

Sensory deafferentation can be considered as an* in vivo* homeostatic plasticity paradigm. An area of cortex is deprived of its normal driving input. As a result, neural plasticity may occur in the form of cortical reorganization as neurons obtain new sources of driving input. However, the case is more complicated than homeostatic plasticity occurring in primary cell cultures as spike timing dependent plasticity must also play a role. In the cortex, neurons are part of a cortical system and the connections or potential connections with other cortical neurons as well as thalamocortical connections serve as infrastructural constraints in the determination of whether reorganization will occur or not. As will become apparent, reorganization can only occur if deprived cortex has access to alternate sources of inputs either through existing subthreshold input pathways or via adjacent cortical areas.

The primate hand is innervated by the median, ulnar, and radial nerves, whose inputs occupy adjacent territory in the somatosensory cortex [27]. Immediately after the median nerve is transected, the cortical territory formally occupied by inputs conveyed by that nerve is largely unresponsive. However, discrete patches of cortex immediately express novel receptive fields innervated by the intact ulnar or radial nerves [20, 28]. The immediacy of the formation of novel receptive fields has generally been supposed to preclude the possibility that completely new synaptic connections have been established. Instead, the “unmasking” of existing but latent inputs has generally been accredited for the appearance of the novel receptive fields [20]. Within weeks to months after median nerve transection, the remaining deprived cortex becomes responsive to stimulation of skin surfaces with intact innervation. Thus, cortical reorganization proceeds as neurons in areas of cortex deprived of input come to express new receptive fields.

Digit amputation in primates also induces somatosensory cortical reorganization [29]. Two months after the amputation of the third digit in an owl monkey, neurons in the cortical area formerly driven by inputs from the third digit respond to stimulation of adjacent digit tips, the palmar pad, and the third digit stump. The cortical representations of adjacent digits 2 and 4 were enlarged by 165% and 180%, respectively. In contrast, the areas of digits 1 and 5 representations showed little change. Amputation of multiple digits also results in the expansion of the representation of adjacent skin areas. However, a silent zone of cortex remains for at least months following digit amputation, suggesting that cortical reorganization is incomplete.

In order to determine if amputation or simply the act of depriving an area of cortex of both primary and latent inputs results in failure to reorganize completely, either the radial and median nerves or radial and ulnar nerves were transected in squirrel monkeys. This procedure deprives digits of both dorsal and glabrous sensory input, mimicking the effects of amputation. After 3–11-month survival periods, large zones of unresponsive cortex remained. Depriving both the volar and dorsal digit surfaces prevents complete reoccupation of deprived cortex by new inputs [30]. The results clearly suggest the presence of dominant and latent inputs to the cortex. Radial nerve dorsal inputs seem to have “preferential access” to glabrous territory innervated by the median and ulnar nerves. Removing radial nerve input prevents reorganization by removing latent inputs. The existence of silent zones following digit amputation [29] or deprivation of both the dorsal and volar hand surfaces [30] illustrates that homeostatic mechanisms alone do not guarantee the establishment of new somatotopic ordering. Rather, there needs to be interplay between homeostatic and spike timing-dependent forms of plasticity that results in reorganization. Moreover, patterns of reorganization after peripheral nerve injury or digit amputation appear to be predicated on preexisting anatomical infrastructure and not on intrinsic cortical properties [30].

Experiential plasticity has also been demonstrated in primates. Owl monkeys were trained to touch the tip of the second digit to a rotating disk with grooves providing 20 Hz tactile stimulation for 3-4 months. Cortical recordings revealed an expansion of second digit territory from 0.32 to 0.92 mm^2^, an almost three-fold expansion. The expansion was limited to the digit stimulated and required that the subject be attending to the task-driven stimulation. Slight decreases in the areas of other digit representations was observed, but the hand representation as a whole also increased in size, expanding into the adjacent cortical area 3a as well as into the representation of the face [31]. This type of experiential plasticity is fundamentally different from deafferentation induced plasticity. Depriving an area of input leads to a diminished area of the cortical representation, while repeated stimulation leads to an increase in the area of the cortical representation. The increase came at the expense of area from neighboring cortical representations.

Cortical reorganization has also been observed following transection of the infraorbital nerve (ION) in the rat somatosensory cortex. Adults (60 days old) and neonates (0 days old) both underwent ION transection and survived for 60 days. In neonates, electrophysiological mapping revealed that the barrel cortex was occupied caudally by whiskers over the eyes and ears, as well as a cheek, ear, and neck representation, and more rostrally by the lower jaw and digits. Reorganization extended 1.5 mm in the rostral portion of the barrel and about 3 mm in the caudal portion. Only a small 0.5–1 mm by 2 mm portion of unresponsive cortex remained near the middle of the barrel cortex. Reorganization in the adult barrel cortex was far less extensive. Sixty days after ION transection, there was a large 3.5 by 2 mm zone of unresponsive cortex. Nonetheless, a 1 mm expansion of cortex responding to stimulation of the whiskers by the ear or over the eye extended into the barrel cortex caudally, while the lower jaw and digit representations extended into the rostral portion of the barrel cortex by about 0.5 mm. Ear and neck representations were also present caudally in the adult barrel cortex [18]. Thus, plasticity appears to be limited in the adult barrel cortex following ION transection, though a relatively small degree of reorganization is present. One possible reason for the difference between juvenile reorganization and adult reorganization following ION transection is the pruning of thalamocortical synapses during the critical period of development. Latent inputs may be present in neonatal rats but lacking in adults. This would further illustrate that, in deafferentation-induced plasticity, homeostatic mechanisms may be necessary but are not sufficient for reorganization to occur. Spike-timing dependent mechanisms appear to also be necessary and they require alternative sources of input in order for temporal correlation to occur. Alternatively, differences in receptor subunit compositions between neonate and adult neurons also exist. Thus, as suggested earlier, immature and mature neurons may be differentially susceptible to homeostatic mechanisms.

In any case, the unmasking of latent inputs following sensory deprivation appears to be immediate and occurs simultaneously (at least in rat) in the cortex as well as in subcortical structures. Simultaneous recordings from adult rat brain stem trigeminal spinal tract nucleus (SpV), thalamic ventral posteromedial nucleus (VPM), and somatosensory cortex (SI) were made before and after some of the whiskers were anesthetized by an injection of lidocaine. After only 3–5 min following lidocaine injection, neurons that previously responded to stimulation of whiskers affected by lidocaine began to respond to the stimulation of neighboring whiskers. No sequence of changes in neuronal receptive fields could be determined as neurons from SpV, VPM, and SI all responded to novel receptive fields simultaneously. The authors hypothesize that the unmasking of alternate receptive fields reflects changes in the balance between excitation and inhibition that occur both cortically and subcortically immediately following the loss of sensory input [32].

Unmasking could be considered homeostatic plasticity that occurs at the circuit level. Receptive fields are refined by afferent driven inhibition. Excitatory neurons have synapses onto inhibitory GABAergic neurons that serve to limit the spread of excitation from sensory stimulation. In the absence of an excitatory drive, inhibition is also reduced, allowing for plasticity to occur between horizontal connections. Although an emphasis has been placed on spike-timing dependent plasticity, there is evidence that homeostatic mechanisms enhance excitability in recently deprived sensory cortex. Adaptations include a release from inhibition and strengthening of glutamate transmission, in some respects similar to mechanisms responsible for enhanced excitability in A1 auditory cortex following ablation of cochleae.

## 4. Homeostatic Mechanisms of Cortical Reorganization

GABAergic neurons are the primary source of inhibitory tone in the somatosensory cortex. Many studies suggest a decrease in GABAergic transmission following sensory deprivation through nerve transection. Glutamic acid decarboxylase (GAD) metabolizes glutamate to form GABA and is thus a marker for GABAergic neurons. In rats, transection of the sciatic nerve followed by a 2-week survival period was accompanied by a 16% decrease in the number of detectable GAD-positive cells staining in layer IV only, with other layers maintaining similar concentrations of GAD-positive cells as found in intact rats. These results suggest that expression of GAD in layer IV varies inversely with the degree of sensory input [33]. In squirrel monkeys, transection of the median and ulnar nerves decreased the number of GABA-positive cells by almost 75% in deprived cortex in comparison to adjacent or ipsilateral nondeprived cortex [21]. Taken together, these studies suggest that a decrease in inhibitory tone may be responsible for the immediate unmasking of latent sensory inputs that occurs immediately following sensory deprivation. An initial decrease in GABA transmission is accompanied by decreases in enzymes involved in GABA metabolism. The immediacy of unmasking suggests that homeostatic mechanisms may operate at the circuit or systems level. Thalamic input drives cortical inhibitory GABAergic input leading to afferent-driven inhibition. Removing excitatory thalamic input also diminishes inhibition. Unmasking, however, has a cellular component as well.

Receptor autoradiographic studies have also shown a reduction in the number of transmembrane GABA receptors in deprived cortex after median and ulnar transection in adult squirrel monkeys. Two to five hours after nerve transection, a decrease in both GABA_A_ and GABA_B_ receptors was present throughout all layers of cortex. However, a decrease in GABA_A_ in layer IV of deprived somatosensory cortex was greater than for other layers of cortex [34]. Reductions in GABA_A_ receptor binding in layer IV are still present 1-2 months after nerve transection. The percent reduction of GABA_B_ receptor binding in layer IV almost doubles over 1-2 months in comparison to reductions observed 1-2 days after nerve transection [35]. Taken together, these studies reveal that GABAergic transmission is diminished directly after nerve transection in terms of both GABA metabolism and the number of GABA receptors.

Although decreases in inhibitory neurotransmission may explain the immediate unmasking of latent inputs, changes in excitatory neurotransmission mediate protracted reorganization of the somatosensory cortex following deafferentation. These changes are reminiscent of spike-timing dependent hippocampal LTP (e.g., [36]), in that they are NMDA receptor dependent ([28, 37]; see [38] for a brief review) and involve an increase in cell-surface AMPA receptors [35]. Blockade of NMDA receptors by the competitive antagonist CPP resulted in only 25% of deprived squirrel monkey somatosensory cortex in area 3b regaining responsiveness after median nerve transection. However, because the administration of CPP was systemic, it is possible that CPP could have acted at subcortical as well as cortical sites to inhibit protracted reorganization. Interestingly, this treatment also diminished cutaneous responsiveness in area 1 for both deprived and nondeprived cortical areas [37]. The role of NMDA receptors is clarified in a subsequent report. Maintenance of reorganization in the somatosensory cortex is not dependent on NDMA receptors. The blockade of NMDA receptors after reorganization has occurred does not cause reorganized cortex to become unresponsive to stimulation of new receptive fields. The initiation of protracted reorganization is NDMA receptor dependent while neither immediate unmasking nor maintenance of reorganized cortex requires the participation of NDMA receptors [28].

Receptor autoradiography reveals an increase in AMPA receptors in layer IV of deprived cortex 1-2 months following median and ulnar nerve transection in adult squirrel monkeys but not 0–3 days after transection. The time course suggests that a change in the number of cell-surface AMPA receptors coincides with protracted reorganization but not immediate unmasking of latent inputs. In contrast, the number of NMDA receptors did not significantly change during either 0–3 days or 1-2 months following nerve transection [35]. This observation stands in contrast to plasticity of input-deprived auditory cortex which includes an increase in EPSC contribution from NMDA receptors [13]. The subunit composition of AMPA receptors was investigated in following reports employing immunohistochemical staining. The calcium permeability of AMPA receptors depends on the presence or absence of the GluR2 subunit, which renders the receptor impermeable to calcium [39]. In a departure from hippocampal-like LTP, one week after median nerve compression in adult squirrel monkeys Mowery and Garraghty [14] observed a decrease in immunohistochemical staining for GluR2/3 subunits and a concurrent increase in GluR1 staining in sensory deprived cortex, with the greatest increase occurring in layers II/III. However, one month after nerve compression an increase in GluR2/3 subunits is observed with no difference in GluR1 expression between deprived and control hemispheres. The results may recapitulate AMPA subunit ratios seen in the developing somatosensory cortex [4]. Developmentally, an increase in GluR2 containing AMPA receptors is observed in rat layer V pyramidal neurons between postnatal days (P) 13-P15 and P16-P21 [40]. In the initial unmasking phase, where much of deprived cortex is unresponsive to cutaneous input, a shift in the ratio of GluR1 to GluR2/3 subunits in favor of GluR1 would enhance calcium permeability and increase calcium-dependent forms of synaptic plasticity. After initial reorganization has been observed 1 month following nerve injury, the increase in GluR2/3 subunits could serve to stabilize synapses and enhance synaptic efficiency, reinforcing the synaptic strength of new and previously underutilized synapses 1 month following nerve injury.

Cortical reorganization shares hallmarks of homeostatic plasticity. Cellular mechanisms, such as the retraction of GABA receptors, act to maintain constant firing rates after principle inputs are lost. Mechanisms of use-dependent plasticity are also altered in favor of strengthening active synapses by increasing cellular calcium permeability. These changes are well-documented examples of homeostatic principles at work.* In vitro* examples of homeostatic plasticity are also present, but their relevance to* in vivo* occurrences is questionable.

## 5. *In Vitro* Homeostatic Plasticity Experiments

Homeostatic plasticity has been examined in cell culture, with the aim of demonstrating that activity regulation is a property of single neurons. Whole-cell recording in response to the application of 0.5 mM glutamate was used to measure miniature excitatory postsynaptic potentials (mEPSPs) from rat visual cortical pyramidal neurons grown in primary cell culture. When cells were grown in the presence of tetrodotoxin (TTX), a potent blocker of voltage-gated sodium channels, neural activity is abolished. The average mEPSP amplitude increased to 192 ± 16% compared to controls. A similar facilitation was seen when neurons were grown in the presence of the AMPA receptor antagonist CNQX. However, growing neurons in the presence of the NMDA receptor antagonist AP5 did not significantly alter mEPSP amplitude. Culturing cells in the presence of the GABA antagonist bicuculline initially increased firing rates to 265 ± 10% of control values, but over a period of 48 h mEPSP amplitudes decreased to 70 ± 4% of controls [15]. Postsynaptic responses to glutamate acted to oppose the perturbations in the system in which neurons were grown. When cultured in the presence of compounds which inhibit cell firing (TTX or CNQX), a larger postsynaptic response to glutamate was observed. When cultured in a condition that enhanced cell firing by inhibiting GABA transmission, the postsynaptic response to glutamate was weakened. In cell culture, the action of perturbing the neuronal environment leads to a reaction in which excitation is modulated in order to oppose external changes. This type of synaptic modulation has been termed synaptic scaling as it preserves relative synaptic strengths established by Hebbian modifications but alters the total synaptic strength of the neuron in order to maintain a global balance of excitation and inhibition. While synaptic scaling may well occur* in vivo*, this experiment fails to recapitulate* in vivo* conditions. The use of TTX is an unnatural manipulation as neurons are never silenced to the same degree or by the same mechanism* in vivo*. The results may have been more relevant to* in vivo* conditions if GABA had been used in place of TTX.

Homeostatic mechanisms have been reported to encompass both presynaptic and postsynaptic loci. The size of the synapse grows when hippocampal neurons are pharmacologically silenced for several days. When hippocampal neurons were cultured in the presence of TTX, the size of the active zone increased from 51 nm to 67 nm and is associated with a size-related increase in the ready release pool and docked vesicle pool sizes. Thus, under chronic disuse, synapses gain strength by releasing more neurotransmitter as a function of an enlargement of the active zone [41]. However, this experiment also relies on TTX, raising questions regarding* in vivo* enlargement of synaptic zones. Prolonged activity blockade also increases postsynaptic density of AMPA receptors at excitatory synapses. Immunolabeling revealed a higher intensity for both GluR1 and GluR2 subunits on the cell surface of cultured visual cortical neurons grown with TTX for 2d [42]. At the synapse, both presynaptic mechanisms leading to an increase in the amount of transmitter released and postsynaptic mechanisms increasing the number and sensitivity of glutamatergic receptors are responsible for increased excitation when neurons are pharmacologically silenced by TTX. Some postsynaptic mechanisms have been confirmed* in vivo*, but the use of TTX confounds comparisons to* in vivo* processes.

Neurons also have the ability to alter their conductance in the face of changing inputs in order to maintain intrinsic firing patterns [43]. Stomatogastric ganglion (STG) neurons from the spiny lobster,* Panulirus interruptus*, usually fire in bursts of action potentials when released from inhibition. When isolated in primary cell culture, neurons fire tonically when depolarized or released from hyperpolarization. However, after 3-4 days of isolation in culture, these neurons fired in bursts when released from hyperpolarization. Yet, if these neurons are supplied with an exogenous rhythmic hyperpolarizing current, mimicking* in vivo* conditions, they lose their ability to endogenously produce burst firing and revert back to tonic firing when the rhythmic stimulation is removed. These experiments show that the STG neurons change their intrinsic conductance in response to changing inputs. However, neurons in isolation are also a deviation from natural conditions. The experiment shows that it is possible for neurons to change their intrinsic conductance but does not demonstrate that this actually occurs* in vivo*.

In the experiments reviewed thus far, homeostatic plasticity has been demonstrated* in vitro* and to a lesser extent* in vivo*. In each case, a neuronal system was perturbed by either increasing or decreasing excitation. The perturbation resulted in a reaction which opposed the perturbation, either decreasing or increasing the excitability of the affected neurons. Homeostatic plasticity occurs in concert with Hebbian forms of plasticity to preserve the transmission and storage of information within the brain. Hebbian plasticity encodes information by strengthening or weakening synapses while homeostatic plasticity preserves information by maintaining neuronal excitability within a physiological range.

## 6. *In Vitro* Use-Dependent Plasticity Experiments

Long-term potentiation (LTP) and long-term depression (LTD) have been evoked in coronal slices prepared from Long-Evans rat somatosensory cortex and studied in synapses of layer IV neurons onto layers II/III. Cells were voltage-clamped at −75 mV and repeatedly depolarized to 0 mV or −50 mV 50–75 times at a constant rate. Evoked EPSCs were found to have increased in amplitude 1.45 ± 0.19-fold from baseline with complete depolarization. Evoked EPSCs decreased in amplitude from 17.1 to 11.2 pA following repeated depolarization to only −50 mV. In both cases, the changes in EPSC amplitudes were stable for at least 30 min [44]. For individual synapses, strong postsynaptic depolarization favors LTP while weak postsynaptic depolarization favors LTD.

Spike-timing dependent plasticity was next examined. EPSCs were evoked at a rate of once per 7.5 s and action potentials (APs) were evoked by injecting current through the recording electrode. When a 3 ms delay was used from the EPSC peak to the postsynaptic AP peak, robust LTP was observed. When a delay of 107 ms was used, with the postsynaptic AP peak preceding the EPSC spike, robust LTD was observed. In terms of temporal summation, when the EPSC occurred slightly before the peak of the AP during the depolarization phase, the net depolarization was greater than when the EPSC occurs during the repolarization phase of the postsynaptic AP. Different delay times were tested in order to explore the temporal limits of spike timing-dependent plasticity. LTP was only induced when the EPSC preceded the AP by 3–14 ms. In contrast, LTD was induced when the AP preceded the EPSC by up to 100 ms, a longer window by almost an order of magnitude [44]. These observations emphasize that the strength of depolarization is dependent upon the timing of EPSPs with APs. When an EPSC overlaps with an AP, the degree of depolarization is greater due to temporal summation.

In order to examine the effect of uncorrelated activity, random delay times between 0 and 50 ms, with either the AP or EPSC occurring first, were employed. These conditions reliably produced a LTD-like decrease in EPSC as predicted by the longer window for LTD induction. Finally, when spike timing-dependent plasticity was attempted in the presence of 50 *μ*m APV, a potent NMDA inhibitor, neither LTP nor LTD could be induced, suggesting that the spike-timing dependent plasticity observed in the somatosensory cortex is NMDA receptor-dependent. Although the majority of the experiments were performed in the presence of BMI to block inhibitory postsynaptic potentials (IPSPs), LTP and LTD were still evoked by similar spike timing procedures in the absence of BMI [44]. These results illustrate that Hebbian forms of plasticity encode information by adjusting the signal-to-noise ratio. If two events occur with temporal or spatial correlation, then these events may be related to one another and the postsynaptic sensitivity to future events is increased. However, if two events are uncorrelated, the events may be unrelated and postsynaptic sensitivity to future events is decreased. In this way, Hebbian plasticity acts as a “molecular coincidence detector.”

In comparing homeostatic to Hebbian plasticity, it is important to realize that Hebbian plasticity is a local and not a global phenomenon. Different synapse of the same neuron can undergo either LTP or LTD independently of each other. Hebbian plasticity alters the relative strengths of individual synapses. Homeostatic plasticity, on the other hand, is a global phenomenon in which the absolute strengths of all synapses of a single neuron are altered or “scaled,” but relative synaptic strengths are preserved.

However, unlike sensory deprivation in the gerbil auditory cortex, sensory deprivation has been shown to evoke LTD in the barrel cortex of Long-Evans rats' somatosensory cortex. The majority of a row of whiskers were plucked from the right side of the whisker pad every other day for 10–20 days. Coronal slices were prepared such that a slice contained one barrel from each row. Field potentials from layers II/III were recorded in response to extracellular stimulation of layer IV. The one-to-one correspondence between whisker and barrel makes the barrel cortex an ideal construct to study deprivation effects of individual whiskers. On every slice, evoked field potentials were about 17% smaller in amplitude in rows of plucked whiskers in comparison to both barrels from intact rows in the same slice and two barrels in the same row that did not undergo deprivation. To confirm these results, excitatory postsynaptic potentials (EPSPs) were measured in whole cell recordings. Threshold currents for which extracellular stimulation in layer IV produces an EPSP in layers II/III were determined and input-output plots were generated for the range 1.0–1.5x threshold and in 0.1x threshold increments. Deprived barrels showed reduced EPSPs compared to intact controls at 1.2–1.5x threshold intensities [45].

Temporal summation predicts that LTD would occur in barrels from plucked whiskers. When the whisker is intact, the activity of layer IV neurons temporally correlates with the activity of layer II/III neurons. In the absence of driving input from the whisker, activity in layers IV and II/III is driven by horizontal rather than vertical connections. Neuronal activity in layers IV and II/III become uncorrelated, and as a result, LTD is induced. Since Hebbian plasticity is a local phenomenon, this does not mean that layers II/III neurons have become less excitable. It only means that layer IV has become less efficient in driving layers II/III neurons.

## 7. Conclusions

In the case of cortical reorganization following sensory deafferentation, homeostatic plasticity might allow for use-dependent plasticity to occur. GABA transmission decreases following the loss of driving inputs, making neurons more excitable. The reduction of calcium impermeable AMPAR subunits may also be a form of homeostatic plasticity, as increased intracellular calcium would also increase neuronal excitability. Yet, these adaptations stretch the definition of homeostatic plasticity if they are labeled such. Much work remains to be done to reconcile homeostatic and use-dependent plasticity.

Fundamental differences exist between* in vitro* and* in vivo* homeostatic plasticity paradigms.* In vitro*, homeostatic experiments have tended to be monosynaptic. This design does not permit measuring the strengths of multiple synapses. To directly reconcile homeostatic and use-dependent plasticity, one would need to show that after LTP occurs at a specific synapse, LTD occurs at other synapses in order to maintain a balance in excitatory drive. Although experiments suggest that synaptic scaling occurs (e.g., [15]), they have not conclusively shown that LTP leads to a decrease in the strength of inactive synapses. Moreover, in living brain tissue, neurons are never completely silenced as they are when cultured in the presence of TTX. There is also no neuropathology resembling such a manipulation. The use of TTX calls into question the relevance of these experiments to* in vivo* brain cells. More work is needed to show that cellular processes studied under the extreme conditions imposed by the use of TTX are exemplars of how neurons behave in the brain.

*In vivo*, more stringent definitions of homeostatic plasticity are needed in order to preserve the meaning of the term. As it stands, any increase in neuronal excitability following the removal of primary driving inputs is considered homeostatic plasticity. However, since this increased excitability is the result of circuit-level changes, such as reduction of afferent-driven inhibition, it is unclear if these changes should be considered homeostatic changes. That is, the focus shifts from individual neurons to systems. Homeostatic plasticity seems to occur, but more* in vivo* research is needed to determine mechanisms.* In vitro* research is also needed but should better conform to conditions that might occur naturally* in vivo*.

Finally, it must be recognized that different tissues (e.g., hippocampus versus cerebral cortex) may differ fundamentally in many important regards due to both evolutionary (i.e., hippocampus is far older) and functional reasons. Thus, hippocampal and cortical neurons may well face different challenges equipped with different mechanisms. Maturity also cannot be overlooked. The mechanisms of plasticity discussed here may themselves be developmentally regulated, and, thus, not necessarily equally efficacious across development. These considerations are no doubt of importance and should be taken into account when results are interpreted.
